# A Respiratory Motion Prediction Method Based on LSTM-AE with Attention Mechanism for Spine Surgery

**DOI:** 10.34133/cbsystems.0063

**Published:** 2024-01-05

**Authors:** Zhe Han, Huanyu Tian, Xiaoguang Han, Jiayuan Wu, Weijun Zhang, Changsheng Li, Liang Qiu, Xingguang Duan, Wei Tian

**Affiliations:** ^1^ School of Medical Technology, Beijing Institute of Technology, Beijing, China.; ^2^ School of Mechatronical Engineering, Beijing Institute of Technology, Beijing, China.; ^3^ Ji Shui Tan Hospital, Beijing, China.; ^4^ Department of Radiation Oncology, Stanford University, Stanford, CA, USA.

## Abstract

Respiratory motion-induced vertebral movements can adversely impact intraoperative spine surgery, resulting in inaccurate positional information of the target region and unexpected damage during the operation. In this paper, we propose a novel deep learning architecture for respiratory motion prediction, which can adapt to different patients. The proposed method utilizes an LSTM-AE with attention mechanism network that can be trained using few-shot datasets during operation. To ensure real-time performance, a dimension reduction method based on the respiration-induced physical movement of spine vertebral bodies is introduced. The experiment collected data from prone-positioned patients under general anaesthesia to validate the prediction accuracy and time efficiency of the LSTM-AE-based motion prediction method. The experimental results demonstrate that the presented method (RMSE: 4.39%) outperforms other methods in terms of accuracy within a learning time of 2 min. The maximum predictive errors under the latency of 333 ms with respect to the *x*, *y*, and *z* axes of the optical camera system were 0.13, 0.07, and 0.10 mm, respectively, within a motion range of 2 mm.

## Introduction

### Research motivation

As a branch of orthopedic surgery, spine surgery is considered to be one of the riskiest and most challenging surgical operations due to the special anatomical structure of the spine and its vital protective role in the central nervous system [1]. In spine surgery, traditional free-hand operations lack guaranteed positioning, which can result in serious complications such as neurological injury, dural sac injury, vascular injury, and so on. Examples of issues that may arise include lateral slip of the casing during the operation [2], irregular displacement of the vertebral body due to pressure from bone or muscle tissue during drilling [3,4], and lateral slip displacement caused by insufficient fixation of the bed frame [3,5].

Robot-assisted technology [6,7] can help surgeons perform operations with greater accuracy and intuition [8], greatly enhancing safety. Various spine surgery robot products have been developed to achieve precise positioning. Most of these products are based on medical imaging systems [including preoperative computed tomography (CT) and intra-operative cone-beam CT (CBCT)] to assist with pre-operation planning and provide surgeons with information about the spine before the operation. Calibration between the CT image frame and the optical camera frame is conducted before the operation, with the relationship represented using a rigid transformation matrix [9].

Modern spinal systems such as Mazor X [10], ROSA spine [11], and TINAVI [12] usually utilize this framework to obtain target vertebral bodies’ position. However, the motion measurement can be indirect, although optical trackers are fixed with spine clamps. One reason for this is that the pose of the tracker recorded before planning is used as the target when the robot moves. However, the time between data recording and robotic motion execution can lead to position errors due to the existence of respiratory motion [13].

Integrating respiratory motion prediction into the spinal surgery robot pipeline can help mitigate the impact of physiological movements. On the one hand, for conventional spine surgery, the prediction in respiratory gating helps to eliminate the time delay that may lead to accuracy loss [14]. On the other hand, robot-assisted decompressive laminectomy requires real-time and predictive information to compensate for the target’s periodic motion, avoiding unexpected contact or collisions [15].

### Related works

The objective of the related work is to improve the accuracy of robotic-assisted pedicle screw placement by addressing tracking errors caused by time latency [16,17]. By employing the mentioned prediction method, robots can achieve more precise screw placement, leading to a reduction in complications. While the majority of studies on respiratory motions primarily focus on tracking lung or liver tumors in radiotherapy to compensate for system latencies [18], a similar approach can be applied to track vertebral segments affected by respiratory motions. Some studies utilize non-invasive surface registration methods to track breathing motions [19]. However, this approach is notably impacted by measurement noise since the measurements are indirect. Alternatively, other studies employ depth sensors to capture the movement of the abdomen or chest during respiration [20,21]. After acquiring the measurements, prediction methods are employed to minimize tracking errors. Prediction techniques such as ARIMA (autoregressive integrated moving average), SVR (support vector regression), and LSTM (long short-term memory) are commonly utilized and can be deployed in real-time applications. These methods enable the system to predict and compensate for motion, thereby enhancing the accuracy of robotic-assisted pedicle screw placement.

Respiratory motion can induce shifts and deformations in the patient’s spine, leading to operation errors of approximately 2 to 3 mm [22]. To tackle this challenge, reliable algorithms for respiratory motion detection and prediction have been developed and verified. Aimed at robot-assisted decompressive laminectomy, a respiration-spine model has been proposed, which takes into account human morphology and ventilator parameters for anesthetized patients. This model enables real-time estimation of spinal fluctuation by measuring gas flow and pressure in the ventilator [15]. A sophisticated nonlinear mechanical ventilation model is presented, which includes the morphometry-based symmetrical structure of the 23 airway generations, dynamic properties of the respiratory system, and a ventilator description [23].

However, the prediction methods mentioned above rely on the inherent data of the ventilator and indirect measurement of respiratory motion of the object areas [24,25], which may be greatly impacted by observation noise.

Prediction approaches are crucial for reducing errors caused by the time delay of detection, as they can provide estimations or regression dynamics to predict intervertebral movement after several time steps, such as the sliding window approach [26]. During thoracic-abdominal puncture (TAP) surgery, a respiratory follow-up robotic system (RFRS) has been developed to ensure accurate surgical puncture. The RFRS utilizes real-time kinesthetic analysis of breathing motion to guide the movement of the robotic arm, which consists of follow-up compensation related to respiratory motion and insertion toward the target [27]. Novel time-varying dual Fourier series have been proposed to model the quasiperiodic cardiac motions. To estimate the series parameters, an extended Kalman filter (EKF) is utilized.

However, regulating the parameters of Kalman filters is difficult for surgeons, and it often fails to converge within a short time. Current respiratory motion prediction methods rely on ARIMA [28], SVR [29,30], or LSTM [31], which have shown great potentials in time-series prediction tasks [32].

One of the most challenging issues is how to apply advanced time-series prediction methods in surgical procedures. This presents a considerable challenge due to the limited time available for learning and recording data. To overcome this challenge, the network needs to be streamlined and trained on a few-shot dataset [33].

### Contribution and organization of this paper

In clinical spinal surgery, it is necessary to anesthetize the patient and provide artificial ventilation. The ventilator, a mechanical device, regulates the airflow into the patient’s lungs through endotracheal intubation. Since respiration is generated by the ventilator, respiratory motion is periodic, allowing the respiratory rate and phase to be assumed as constant values. However, these parameters need to be estimated during operations. One possible approach is to use a neural network as a regression method to develop a model using actual respiratory motion data. This allows for accurate estimation of respiratory parameters in real time, facilitating precise control and optimization of robotic or surgical systems that depend on respiratory motion.

It is crucial to acknowledge that respiratory patterns and models may differ among various patients. Hence, before each surgery, the neural network needs to be trained using a dedicated database specific to the individual patient. To avoid prolonging the operation unnecessarily, the database should comprise sample points within a 30-s time span. In order to validate the outcomes of the proposed scheme, an experimental database is collected using an optical measurement device. The optical tracker is fixed on the patient’s vertebral body by a spine clamp. This ensures accurate and reliable data collection for training the neural network.

The main contributions of this essay can be summarized as follows:

• A method based on principal components analysis (PCA) is proposed to achieve dimension reduction of respiratory motions.
• A novel network is proposed for predicting the respiratory motion of patients during operation utilizing an LSTM with attention mechanism and an autoencoder (AE) layer for the respiration prediction approach. The network is trained with sample points collected over a duration of approximately 30 s. The method aims to accurately regress and predict the respiratory motion of patients to enable efficient and effective surgery.

The remaining sections of this paper are organized as follows. The “Respiratory Motion Database” section presents the data preprocessing method and establishes the database of respiratory motion for different patients. The “Method” section illustrates the neural network-based prediction frameworks and the corresponding function of each part. The “Experiments and Results” section presents the experimental validation of the proposed scheme using limited respiratory sampling points. Finally, in the “Discussion and Conclusions” section, concluding remarks on this research work are provided.

## Respiratory Motion Database

This section presents a comprehensive explanation of the algorithms used for dimension reduction of respiratory motions. Additionally, a framework for measurement and preprocessing is proposed to establish a respiratory motion dataset for each patient.

### Data collection

**Fig. 1.**
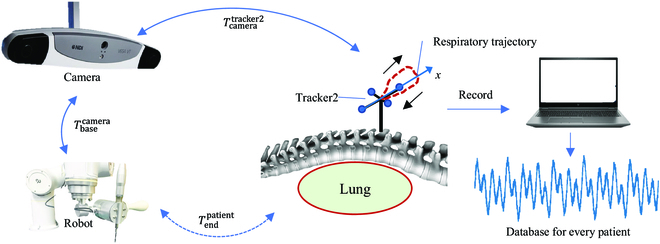
The schematic diagram of robot-assisted spine surgery requires the tracker’s positional information captured from camera, with the data recorded for every patient.

To complete spinal surgery using a robot, 2 crucial steps are involved. The first step is hand–eye calibration, which involves acquiring the matrix between the robot and the camera system. After completing this step, data captured by the camera can be transformed into the robot frame using the following equation:

$$T_{\text{base}}^{\text{tracker}1}=T_{\text{camera}}^{\text{tracker}1}T_{\text{base}}^{\text{camera}}$$ (1)

where $T_{\text{base}}^{\text{camera}}$ is the result of hand–eye calibration and $T_{\text{camera}}^{\text{tracker}1}$ is the motion of robot captured by the optical camera system. The second step is patient registration, which involves obtaining the transformation matrix between the CT image frame and the optical camera frame using the optical tracker. To account for the influence of breathing, the optical tracker can be fixed on the exposed segment using a spine clamp, as depicted in Fig. 1. With Eq. 2, the robot end effector could achieve accurate positioning tasks.

$$T_{end}^{\text{patient}}=T_{\text{tracker}2}^{\text{patient}}T_{\text{camera}}^{\text{tracker}2}T_{\text{base}}^{\text{camera}}T_{end}^{\text{base}}$$ (2)

**Fig. 2.**
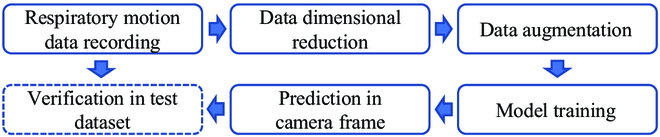
The overall method pipeline comprises data recording, dimension reduction, data augmentation, model training, prediction, and verification.

We assume that the matrices $T_{\text{patient}}^{\text{tracker}2}$ representing the relationship between the patient and the patient’s tracker, and $T_{\text{base}}^{\text{camera}}$ are constant. $T_{\text{end}}^{\text{base}}$ could be obtained using robotic kinematics. Therefore, we can represent respiratory motion by $T_{\text{camera}}^{\text{tracker}2}$ as a simplification. Consequently, only camera devices are required in this scenario, and the $T_{\text{camera}}^{\text{tracker}2}$ recorded at different time steps are considered as the raw data of respiratory trajectories. Using Eq. 2, the robot’s end effector can accomplish accurate positioning tasks.

### Dimension reduction of respiratory motion

Due to the anatomical structure of the spine, the influence of respiratory movement is mainly reflected in a specific axis of the human body. In this study, the respiratory motion data recorded by the tracker is represented by a closed curve situated along an axis, as situated by the red trajectory and *x* axis in Fig. 1. Notably, the tracker first undergoes an upward movement, then moves downward to return to the starting point, which is generated by inhalation and exhalation. To mathematically represent the primary motion axis with respect to the camera frame, we introduce a PCA-based method, which is applied to achieve dimension reduction.

First, the sampling points can be formed as a matrix $X=\left(\begin{array}{llll} x_{1} & x_{2} & \ldots & x_{n}\\ y_{1} & y_{2} & \ldots & y_{n}\\ z_{1} & z_{2} & \ldots & z_{n} \end{array}\right)$ , where the subscripts (1, 2, ..., *n*) represent the chronological serial number within one loop. To determine the primary direction responsible for the most substantial numerical change, it is necessary to use a covariance expression denoted as *Cov*, as shown in Eq. 3:

$$\begin{array}{ll} \mathrm{Cov} & =\frac{1}{n}\left(X-\bar{X}\right){\left(X-\bar{X}\right)}^{T}\\ & =\sum_{k=1}^{3}‍\sigma_{k}v_{k}v_{k}^{H} \end{array}$$ (3)

where $\bar{X}$ is the average value of all sampling points recorded in *X*. The covariance expression can be numerical decomposed into 3 parts, and each part is defined by the product of eigenvector and the corresponding eigenvalue. The eigenvectors are the directions along which the data vary the most, and the eigenvalues represent the amount of variance captured by each eigenvector. The eigenvectors are sorted in descending order of their corresponding eigenvalues, and the eigenvector with the highest eigenvalue represents the first principal component (PC1), the index of it is denoted as $k_{\text{max}}=\underset{k}{\text{argmax}}\sigma_{k}$. Eigenvalues and eigenvetors could be resolved with SVD algorithm. According to the PCA method, the primary axis of respiratory motion can be described as $X_{\text{axis}}=v_{{\text{k}}_{\text{max}}}$. The projection of sampling points onto the axis *X_p_* can be represented via Eq. 4.

$$X_{p}=v_{k_{\max}}^{H}X=\left[x_{p,1},x_{p,2},\ldots x_{p,n}\right]$$ (4)

where *X_p_* is the transformed dataset, *X* is the original dataset, and $v_{{\text{k}}_{\max}}$ corresponds to PC1. The transformed dataset *X_p_* represents a compressed representation of the original dataset *X*, where the data are projected onto the directions of maximum variance in the original data. Any element in the vector *x*_*p*,*k*_ can represent the tracker’s movement caused by respiratory motion.

### Data augmentation for few-shot learning

To address data limitations during surgery, data augmentation is introduced to generate new data associated with respiratory motion from existing data. The augmentation database can improve the regression performance of machine learning-based models by providing new and diverse examples for training datasets. According to Eq. 5, the source of data augmentation comes from the affine transformation of coordinates on *x_p_* axis: Eq. 5

$$x_{\mathrm{new}}=\left(1-\beta \right)x_{p}+\beta y_{p}$$ (5)

where *x_p_* is the original data projected to PC1 and *y_p_* is the original data projected to PC2, which is the second principal component of respiratory motion. The coefficient *β* denotes the offset from *x_p_* axis to *y_p_* axis, and the generated data *x*_*new*_ are then normalized to ensure that the points in the dataset fall within the range from −1.0 to 1.0.

In fact, there may be errors and disturbances in the primary motion axis, and the augmented data simulate the projections on different but approximate axes to the primary motion axis, with the respiratory rate and phase being the same as the existing data. Therefore, the attributes between the existing points and the augmented points are consistent.

## Method

### Network architecture

Respiratory motion is a periodic motion, with one of its most important features being a time-dependent phenomenon. Recurrent neural networks (RNNs) have been proven to be a powerful tool for addressing time-series problems [4]. RNNs have the ability to capture temporal dependencies in time-series data by maintaining a hidden state that carries information from previous time steps to the current time step, allowing them to capture patterns and trends in the data. LSTM is an extension of RNNs that incorporates a gating mechanism to control the flow of information and gradients through the network. This gating mechanism allows LSTMs to selectively update, forget, or output information at each time step, providing greater flexibility in modeling complex sequences compared to traditional RNNs. Specifically, LSTMs are capable of learning from limited training data, making them suitable for few-shot training scenarios. The overall method pipeline is shown in Fig. 2.

**Fig. 3.**
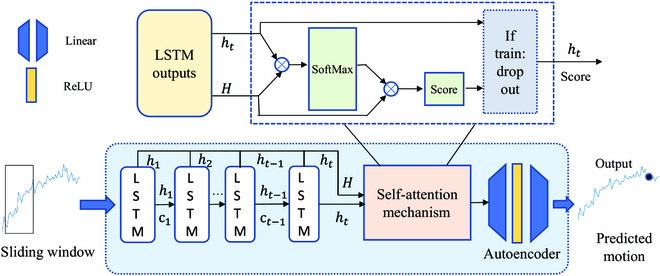
The sampling points within sliding windows are served as inputs for the cascading of LSTMs, self-attention mechanism, and autoencoder, with the outputs being predicted motions.

Our architecture, as illustrated in Fig. 3, incorporates LSTMs with attention mechanism and an AE layer, for respiration prediction. LSTM can maintain a longer-term memory of the past while overcoming the gradient disappearance and gradient explosion problems of long sequence training. The attention mechanism is used to selectively attend to different regions or time steps of the respiratory motion signal, depending on their importance or relevance to the current prediction. The AE network is specifically designed to extract important features of the input data in a lower-dimensional latent space.

### Long short-term memory

In this paper, the inputs for neural network are based on sliding window, which is a segment of consecutive data points extracted from the respiratory motion time series data, while the output represents the position of the predicted point after a certain time interval. The predicted output can be verified with the sampling point corresponding to the same time interval, which is defined as ground truth.

**Fig. 4.**
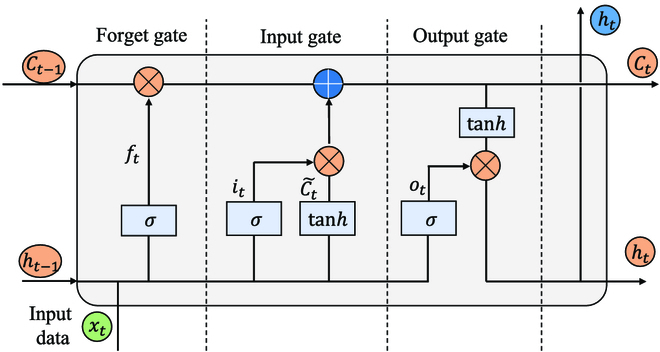
The structure of LSTM network returns *h_t_* and *C_t_* as outputs.

The time interval is 333 ms (10 sampling points apart), and the window size *n* is set to 50, which corresponds to the number of layers in the LSTM network. As shown in Fig. 3 , each point will be input into an LSTM unit according to the chronological order of the points. There are 2 main internal states during the procedure: hidden state and cell state, denoted as *h_t_* and *c_t_*, respectively. Apart from the first point, *h_t_* and *c_t_* combined with each point is used as the input for the next time step of the LSTM and plays a crucial role in the LSTM’s ability to capture temporal dependencies and learn from sequential data. The hidden state is the output of the LSTM unit at the current time step, and the cell state is the memory state of the LSTM unit that is updated at each time step based on the input data and the internal gating mechanisms of the LSTM. The gating mechanism of LSTM shown in Fig. 4 could be described as Eq. 6 to Eq. 11:

Input gate(*i_t_*):

$$i_{t}=\sigma \left(W_{ix}x_{t}+W_{ih}h_{t-1}+b_{i}\right)$$ (6)

Forget gate(*f_t_*):

$$f_{t}=\sigma \left(W_{fx}x_{t}+W_{fh}h_{t-1}+b_{f}\right)$$ (7)

Output gate(*o_t_*):

$$o_{t}=\sigma \left(W_{ox}x_{t}+W_{oh}h_{t-1}+b_{o}\right)$$ (8)

Candidate cell state(${\tilde{c}}_{t}$):

$${\tilde{c}}_{t}=tanh\left(W_{cx}x_{t}+W_{ch}h_{t-1}+b_{c}\right)$$ (9)

Cell state(*c_t_*):

$$c_{t}=f_{t}C_{t-1}+i_{t}\cdot {\tilde{c}}_{t}$$ (10)

Hidden state(*h_t_*):

$$\begin{array}{l} h_{t}=o_{t}\cdot \tanh\left(c_{t}\right) \end{array}$$ (11)

Among them, *W_ix_*, *W_ih_*, *W_fx_*, *W_fh_*, *W_ox_*, *W_oh_*, *W_cx_*, and *W_ch_* represent weight matrices for input, hidden state, forget gate, and candidate cell state, respectively, while *b_i_*, *b_f_*, *b_o_*, and *b_c_* are bias terms for input gate, forget gate, output gate, and candidate cell state, respectively. *σ*(·) represents sigmoid activation function, and tanh(·) represents hyperbolic tangent activation function. *x_t_* is the input at time step *t*.

Notably, the hidden states and outputs are recorded to serve as inputs of attention mechanism model.

### Attention mechanism

The attention module, as depicted in the pink block of Fig. 3, is implemented using the self-attention mechanism. It receives sequences processed by the LSTM and outputs time-independent features for the AE network. The self-attention mechanism operates on a sequence of LSTM-recursive outputs and assigns weights to the sequence, enabling the network to determine the focused subsequence.

In this scenario, the query and key vectors are the hidden states of LSTM layers. The key vector is the final output of LSTM, and query vectors are the entire outputs of every LSTM unit.

The attention scores and values served as the input for the AE network. In addition, value can be represented by output of LSTM. As a result, the expression of attention can be denoted via Eqs. 12a and Eqs. 12b:

$$A=\text{softmax}\left(H_{l}*H\right)$$ (12a)

s=AH (12b)

where the tensor *H* represents the hidden states of LSTM layers, with *H_l_* representing the last LSTM output. The variable *A* is used to help the network focus on important time periods. To achieve normalization and prevent saturation, a softmax operation is performed.

As a result, the integration of LSTM and the self-attention mechanism enhance the network’s ability to predict time sequences with higher time delays.

### Autoencoder

The utilization of an AE can help with extracting important features from the output of the self-attention mechanism, and further reduce the dimensionality of the data.

As illustrated in Fig. 3, the AE consists of an encoder and a decoder. In this paper, the encoder is composed of a linear layer and a rectified linear unit (ReLU) activation function, while the decoder consists of only a linear layer. The encoder maps the extracted features from LSTM with attention mechanism to a lower-dimensional latent space, and the decoder maps the latent space representation back to the original input space. In our case, the size of the sliding window is set to 50, which is designed to cover the majority of the features in a single respiratory cycle.

## Experiments and Results

### System setup

**Fig. 5.**
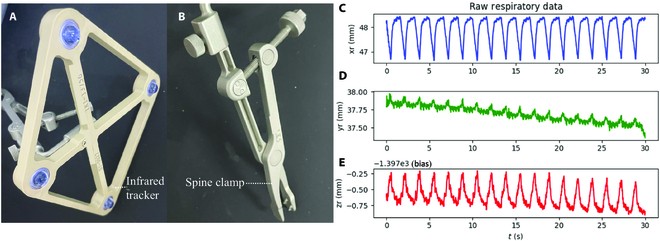
(A) Dedicated tracker used to record the vertebral body’s motion. (B) The spine clamp can be fixed on patient’s vertebral bodies. (C) Recorded respiratory data on *x* axis under camera’s frame. (D) Recorded respiratory data on *y* axis under camera’s frame. (E) Recorded respiratory data on *z* axis under camera’s frame.

In order to evaluate the performance of the proposed framework for respiratory motion prediction, a verification process was conducted. An infrared optical tracking system was utilized in the experiment, which comprised an NDI camera (Passive Polaris Spectra, NDI, Waterloo, ON, Canada) and a dedicated tracker, as shown in Fig. 5. In the clinic, the patient was guided to lie in the prone position, and the camera should be positioned ahead of the patient’s head to avoid obstructing the surgeon’s view. Once the patient is anesthetized, the back skin above the target vertebral segment is cut, and typically, L2 (lumbar 2) and L5 (lumbar 5) vertebral bodies are exposed.

Aiming at lumbar spine indications, surgeons fixed the tracker on L2 vertebral body using a spine clamp.

These vertebral bodies were selected because their anatomical landmarks can be reliably localized, and the respiration-induced movements of these segments reflect the overall tendency of the spine. To meet clinical requirements, the NDI camera operated at a frequency of 30 Hz, and the position changes of the trackers were recorded as a 7-dimensional vector including x, y, z, q0, qx, qy, and qz. The recorded data are saved in the computer as the raw trajectory file. Only one vertebral segment’s motion was monitored at a time while the patient was breathing, with a total recording time of approximately 30 s (900 frames) for each segment. The position of the NDI camera remained unchanged during the measurements.

### Experimental database

The experimental database comprises 4 cases of clinical respiratory movement trajectories, with each case of respiratory movement signal having a data collection duration of 30 s. Figure 5 (C to E) shows the respiratory motion trajectory of one patient in the *x*, *y*, and *z* axes under the camera frame, denoted as *x_r_*, *y_r_*, and *z_r_*, respectively. Furthermore, it should be noted that the patients were under deep anesthesia, and the respiratory motion was solely influenced by the ventilator. The ventilator parameters were illustrated in Table 1.

**Table 1.** Ventilator parameters

Name	Value
*V_T_*	475 (ml)
Rate	12 (/min)
I:E	1:2
Plimit	40 (cm H_2_O)
PEEP	Off
Control mode	Volume control

**Table 2.** The eigenvalues for PC1, PC2, and PC3

Index	Eigenvalue in PC1	Eigenvalue in PC2	Eigenvalue in PC3
Trajectory 1	0.295	0.031	0.002
Trajectory 2	0.288	0.001	0.001
Trajectory 3	0.311	0.050	0.002
Trajectory 4	0.559	0.015	0.005

During the data preprocessing stage, the method described in the “Dimension reduction of respiratory motion” section was employed to process the collected clinical data, resulting in the reduction of the 3-dimensional vector to 1-dimensional vector. Afterward, the method in the “Data augmentation for few-shot learning” section was used to augment the data for training and validation datasets.

**Fig. 6.**
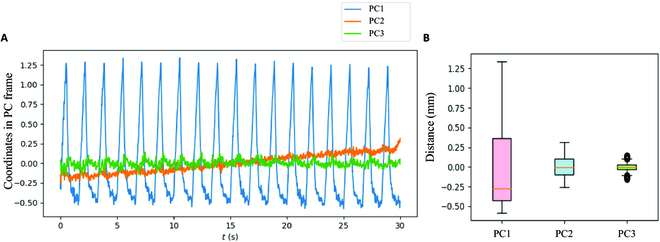
(A) Processed respiratory data on PC1, PC2, and PC3 axes of the frame derived from PCA method. (B) Box-line chart of data dispersion in the 3 axes.

Three eigenvectors were obtained using the PCA method, with the primary motion axis denoted as *n*_*x*1_ = [−9.60 × 10^−1^, 6.34 × 10^−2^, 2.74 × 10^−1^]. Figure 6 A shows the original data projected onto PC1, PC2, and PC3 axes, while Fig. 6 B shows the box-line chart of the 3 axes. It is evident that the changes in PC1 are the largest.

In terms of all the cases, the eigenvalues in PC1, PC2, and PC3 were displayed in Table 2. The motions along the PC1 axis were at least 6 times greater than those along the PC2 axis, and at least 100 times greater than those along the most irrelevant axes. In our experiment, quaternions were chosen for the depiction of orientations, and the highest variations were around 0.02, which could be disregarded as it had minimal impact on the tracked point.

After data preprocessing, every 50 points will serve as input for the sliding window, with the ground truth being selected at every 10th interval.

### Training model

During surgery, it is necessary to develop a respiratory model for each patient to account for individual differences in respiratory motion rate and phase due to physiological variations. Based on the previously explained architecture, a dataset of tracker trajectories is constructed and used for training respiratory models for different patients.

The loss function of the presented network is derived from the L2 Euclidean norm of predictions in Eq. 13:

$$\text{loss}={\left\|e\right\|}_{2}=\|y_{o}-y_{\text{real}}{\|}_{2}$$ (13)

where the loss drives the back-propagation during the training process. *y_o_* represents the network’s prediction value, and *y*_real_ is the ground truth. The learning rate is set to be adaptive to save training time. There are 2 steps in training: the first is to find a suitable region that contains the optimal parameters of the neural network model, and the second is to refine the parameters to improve the network’s performance.

The entire dataset was divided into 3 parts with a ratio of 6:2:2 for the training set, validation set, and testing set, respectively.

**Fig. 7.**
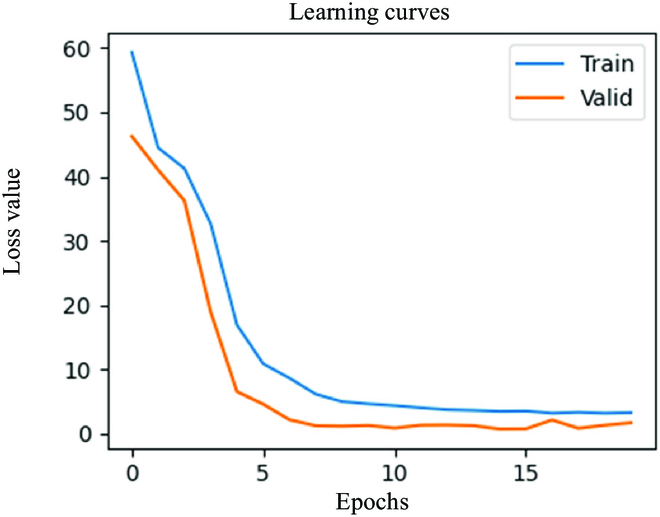
The learning curves on the training set and validation set.

In our implementation, the network’s learning rate was adjusted for the Adam optimizer. We initially set the learning rate to 10^−4^, and then decreased it to 10^−5^ after 5 epochs, resulting in a more stable training update. The training process, as depicted in Fig. 7, exhibited a notable decrease in training loss during the first 5 epochs. We used a sliding window length of *L* = 50 as indicators, and conducted training for 20 epochs, based on empirical results. The minimum loss value was observed at epoch 15, reaching 0.83. The entire training procedure was completed within 2 min using an RTX 3080 graphics card, which meets the time-consuming requirements for clinical applications.

### Results

After obtaining the model, the test set is used for comparing the performance of different methods. The test set only contains raw input–output–pair data rather than augmentation data.

We utilized the root mean square error (RMSE) value and the maximum prediction error to evaluate the performance of the proposed method. Noticeably, RMSE provides a metric of the average performance of the predictions representing the basic quality of algorithms; however, maximum prediction errors served as supplements for the worst-case predicted errors.

**Fig. 8.**
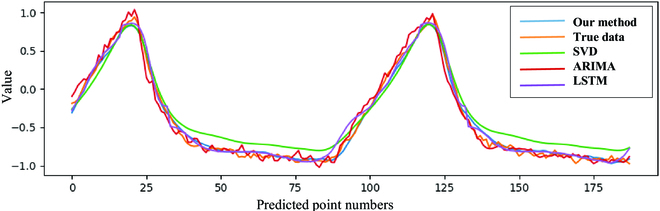
The comparison of various predicting methods for the respiratory motion predicting task under the condition of few-shot dataset usage.

SVR, ARIMA, and simple LSTM were selected as baseline algorithms, and the results are illustrated in Fig. 8 and Table 3.

**Table 3.** Performances of different prediction methods

Name	RMSE (%)	Maximum errors (%)
Our method	4.39	15.07
SVR	12.22	24.51
ARIMA	6.23	25.10
LSTM	5.13	21.45

Additionally, all the methods were capable of real-time implementation, as the time-costing of the forward procedure was less than 10 ms.

Our method exhibited the best performance in respiratory motion prediction, as evidenced by the lowest RMSE and the least maximum error. Additionally, the errors were expressed as percentages, as the raw data had been normalized.

**Fig. 9.**
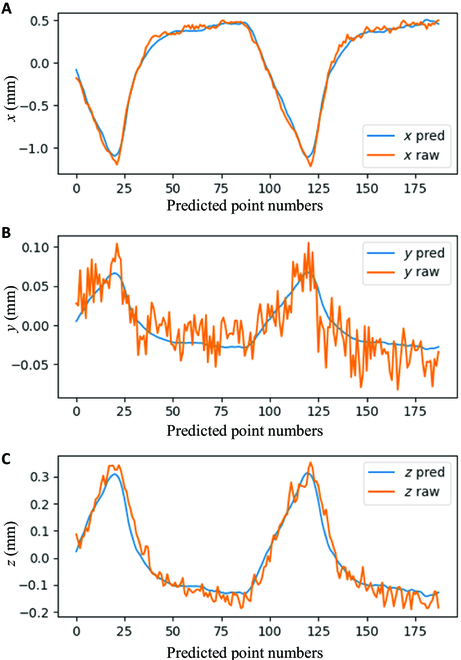
(A) Prediction performance in test set with respect to *x* axis of the camera frame. (B) Prediction performance in test set with respect to *y* axis of the camera frame. (C) Prediction performance in test set with respect to *z* axis of the camera frame.

Given that the maximum range of respiratory motion was within 2 to 3 mm, we were able to calculate the overall predictive error. As shown in Fig. 9, we considered a 2-mm motion range in our cases, and the maximum predictive errors in the *x*, *y*, and *z* axes were 0.13, 0.07, and 0.10 mm, respectively. Consequently, the corresponding overall error in Cartesian space was within 0.5 mm, which aligns with the demands of tracking systems.

## Discussion and Conclusions

In this paper, we present a method that utilizes PCA for dimensional reduction and network regression to predict the respiratory motion of vertebral segments in spine surgery.

Clinical raw data were obtained during surgery, with a recording time of up to 30 s. The data augmentation method was applied to create more training and validation data for the few-shot datasets built from patients’ operations. The respiratory motion was then compressed to a single-dimensional signal using PCA and regressed with a sliding-window-based neural network. Compared to conventional methods such as LSTM, SVR, and ARIMA, this method has a stronger ability to facilitate respiratory motion predictions with fewer datasets and higher prediction accuracy. Finally, it achieved an accuracy of RMSE 4.39% and a maximum error of 15.07%, which is notably better than other methods. The maximum predictive error with respect to the *x*, *y*, and *z* channels of tracking systems was 0.13, 0.07, and 0.10 mm, respectively, which is within the tracking error tolerance of spine surgery.

On the one hand, the method benefits from adjustable learning rates and streamlined network structures, leading to a reduction in learning time to only 2 min. This fast recording and learning ability facilitates the application of the method to clinical systems, allowing surgeons to easily insert the recording or learning procedure into their current surgical pipeline. On the other hand, the predicted range can be extended up to 333 ms with a forward procedure time of less than 10 ms. In practice, information acquired by navigation systems cannot be instantaneously transmitted to the robotic arm control system, resulting in a certain degree of delay. Consequently, the robotic arm cannot achieve real-time and precise positioning and manipulation. The method proposed in this paper can provide respiratory motion predictions within as many as 10 sampling points, compensating for errors caused by delays in control. This equips robot-assisted spinal surgery systems with real-time tracking and adjustment capabilities, enabling them to adapt to a patient’s respiratory motion effectively. Furthermore, the evaluation confirmed the ability of the method to forecast and track respiratory motions in all dimensions of tracking systems (*x*, *y*, and *z*) effectively.

However, the method proposed in this paper has certain limitations. In actual clinical surgeries, it is not feasible to install tracking markers within the target vertebra as it would impede the surgeon’s operating space. Therefore, the analysis of intervertebral micro-motion is one of the key areas of focus for future research in this study. By incorporating intervertebral micro-motion data with real-time motion data from adjacent vertebrae, we aim to comprehensively consider the physiological motion’s impact on the target vertebra, extending beyond just respiratory motion.

## Data Availability

Data are available upon reasonable request.
